# Remarkable tumor response to Iruplinalkib in a 14-year-old girl with *ALK*-positive advanced metastatic non–small-cell lung cancer: a case report

**DOI:** 10.3389/fonc.2025.1645580

**Published:** 2025-07-25

**Authors:** Jiahao Wang, Qingdi Du, Xueyan Sun, Rong Tao, Zuojuan Zhang, Wei Li, Wenjing Liu, Li Wei, Zongsheng Tian, Xueliang Xu

**Affiliations:** ^1^ Department of Respiratory and Critical Care Medicine, Linyi People’s Hospital, Linyi, China; ^2^ Department of Diagnostics and Clinical Medicine, Shandong Medical College, Linyi, China; ^3^ Department of Thoracic Surgery, Linyi People’s Hospital, Linyi, China; ^4^ Department of Pathology, Linyi People’s Hospital, Linyi, China

**Keywords:** non-small cell lung cancer, *ALK* fusion gene mutation, Iruplinalkib, targeted therapy, case report

## Abstract

Targeted therapies for anaplastic lymphoma kinase (*ALK*) mutations in non-small cell lung cancer (NSCLC) generally extend survival and alleviate symptoms. However, significant tumor reduction or complete remission remains rare. We report a rare case of a 14-year-old girl, whose father and grandfather both had lung cancer, diagnosed with advanced, multi-site metastatic *ALK*-positive NSCLC. She was treated with Iruplinalkib, a newly approved targeted therapy in China, resulting in remarkable tumor shrinkage. The patient presented with severe symptoms, including persistent cough, pain, and hemoptysis. A lung CT scan revealed a large mass, which was pathologically diagnosed as pulmonary adenocarcinoma. After initiating Iruplinalkib therapy, the primary tumor rapidly decreased in size by 80.3%, from 132 mm × 97 mm to 26 mm × 21 mm, within one month. Most metastatic lesions also showed significant regression. By six months, the pulmonary tumor had almost disappeared. This case underscores the potential of Iruplinalkib, which is currently not available outside of China, to induce rapid and profound tumor regression in *ALK*-positive NSCLC, particularly in adolescent patients with aggressive clinical presentations. We hope that the anticancer efficacy of Iruplinalkib will be recognized globally and that it will become accessible to *ALK*-positive lung cancer patients worldwide.

## Introduction

According to global cancer statistics, lung cancer accounts for over 2.5 million new cases annually, representing approximately 12.4% of all cancer diagnoses. It is also the leading cause of cancer-related deaths, responsible for around 1.8 million fatalities each year—nearly one-fifth of the global cancer mortality burden (1, 2). In NSCLC, approximately 60% of patients harbor actionable driver mutations, making them eligible for targeted therapies, which have shown superior efficacy compared to traditional chemotherapy and radiotherapy (3). Among these, *ALK* alterations or fusions are relatively rare, occurring in approximately 3% to 7% of NSCLC cases. *ALK*, a receptor tyrosine kinase in the insulin receptor subfamily, is more commonly mutated in young, never-smoking females. The prevalence of *ALK*-positive NSCLC is 5% to 7% in East Asians and 3% to 6% in Caucasians. Notably, in NSCLC patients under the age of 40, *ALK* positivity can reach up to 20% (4). *ALK* signaling in cancer cells is mainly activated through gene fusion, amplification, or point mutations, with *EML4-ALK* fusion being the most common. This fusion leads to continuous *ALK* kinase activity, driving tumor growth and metastasis. Despite its low incidence, *ALK* fusion mutations respond well to *ALK* Tyrosine Kinase Inhibitor (*ALK*-TKI), with a 5-year survival rate over 60% and superior clinical outcomes, earning the nickname “diamond mutation” (5). To date, the The Food and Drug Administration of the United States has approved five *ALK*-TKIs: first-generation crizotinib, second-generation ceritinib, brigatinib, alectinib, and third-generation lorlatinib (6). Compared with chemotherapy, *ALK*-TKI treatment achieves longer progression-free survival (PFS) and higher objective response rate (ORR). However, most targeted therapies only inhibit tumor growth and prolong PFS, with rapid tumor regression or elimination remaining rare (7).

Iruplinalkib is a novel second-generation TKI targeting both *ALK* and *ROS1*, currently approved only in China (8). This study reports a rare case of a 14-year-old patient with advanced, multi-site metastatic NSCLC and a strong family history of lung cancer. Genetic testing confirmed an *ALK* fusion mutation. After six months of iruplinalkib therapy, the patient’s large pulmonary mass had nearly disappeared, demonstrating a remarkable and near-miraculous therapeutic response.

## Case report

**Figure 1:**
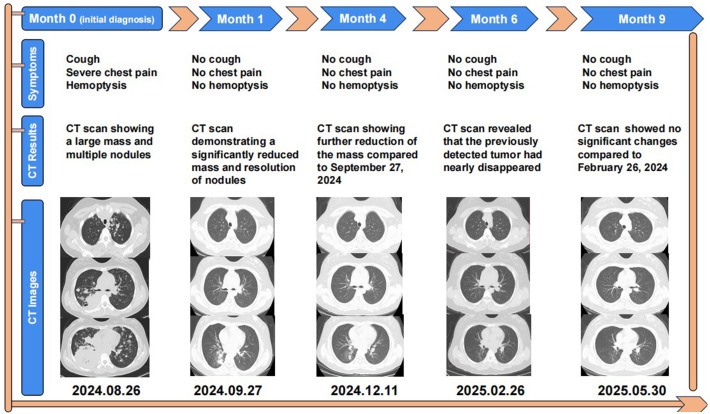
Graphic summary of the case.

A 14-year-old girl, whose father and grandfather both had lung cancer, presented to the respiratory department in August 2024 with weight loss (20kg over 7 months). Her main concerns and symptoms are coughing, hemoptysis, and severe chest pain. The patient had no other medical history prior to this illness and has not undergone any investigations or interventions since the onset of her symptoms. A physical examination showed enlargement of supraclavicular lymph nodes and decreased breath sounds in the right lung, other physical examination findings were unremarkable. Laboratory investigations showed increased tumor markers: CEA 2580 ng/ml, CA125–13692 U/ml, CA19-9–803 U/ml, CA153–685 U/ml, CA72-4–428 U/ml, NSE 138 ng/ml. On August 26, 2024, a CT scan revealed a 132×97 mm mass in the right lower lobe, along with multiple pulmonary nodules, enlarged mediastinal and right hilar lymph nodes, liver nodules, and abdominal lymphadenopathy (**Figure 1**).

After admission, the patient’s symptoms continued to worsen, with severe pain accompanied by massive hemoptysis (100–500 ml per day), and despite treatment with hemostatic drugs and morphine for pain relief, there was no improvement.

**Figure 2:**
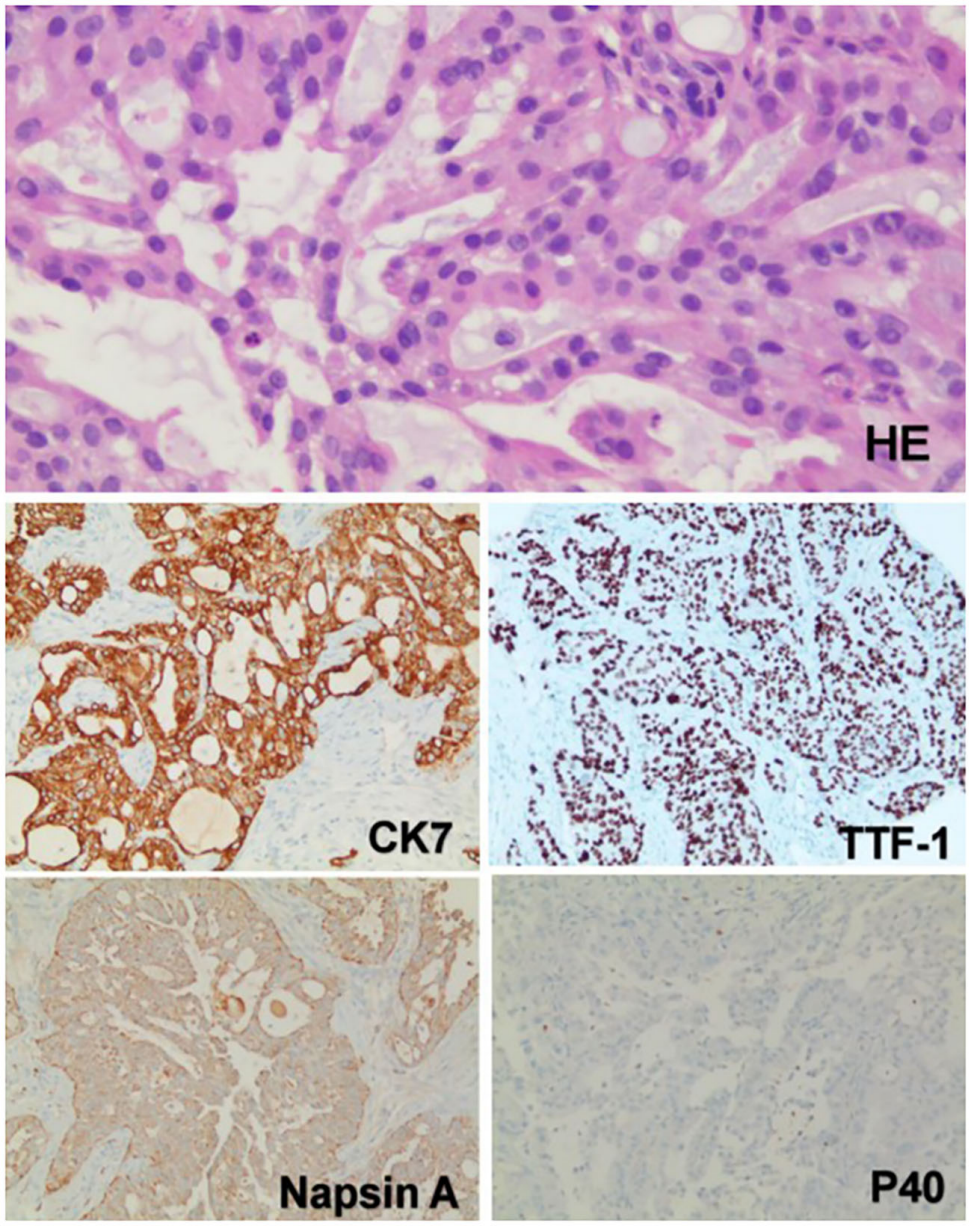
The pathology and immunohistochemistry of the mass in the right lower lobe. Hematoxylin and eosin (HE) staining revealed that the tumor cells were arranged in acinar, nest-like, and solid patterns. Intracytoplasmic mucin was observed in the tumor cells, with focal areas suggesting the presence of signet-ring-like cells. Immunohistochemical analysis showed diffuse positivity for TTF-1, CK7, and Napsin A, indicating a pulmonary origin. The absence of P40 expression excluded squamous cell carcinoma. Based on these findings, the tumor was considered to be a pulmonary adenocarcinoma. (Immunohistochemical staining was performed using the Ventana Medical Systems platform; detailed protocols are provided in the **Supplementary Materials**.).

She underwent CT-guided puncture biopsy of the mass, which showed adenocarcinoma with poor differentiation. Immunohistochemistry was positive for CK7, TTF-1, and Napsin A, but negative for P40, which suggested a lung primary (**Figure 2**). The patient was diagnosed with NSCLC (T4N2M1c), adenocarcinoma with metastases to hilar and mediastinal lymph nodes, liver, bones, and abdominal lymph nodes.

On August 30, 2024, molecular analysis of *EGFR*, *KRAS*, *BRAF*, *ROS1*, *RET*, *PIK3CA*, *MET*, *ERBB2*, *NRAS* revealed absence of mutations. However, *ALK* fusion gene mutation was positive. The genetic testing was conducted using tissue samples. While a more comprehensive liquid biopsy could potentially provide more accurate and complete results (9), regrettably, the patient did not choose this option due to financial considerations. The genetic testing was performed using the ARMS quantitative real-time PCR method (The specific testing procedures and detailed reports, including the exact type of fusion, are provided in the **Supplementary Materials**). Based on the latest clinical research data, Iruplinalkib has shown superior therapeutic efficacy compared to other ALK-TKIs. Additionally, as a newly approved agent, it is currently included in programs offering financial assistance. Given the patient’s critical condition and economic circumstances, she and her family selected Iruplinalkib (starting at 60 mg once daily and increasing to 120 mg once daily after 8 days) as the initial treatment. This treatment plan was reviewed and approved following discussion by a multidisciplinary tumor board. Following 5 days of treatment, her symptoms significantly improved, leading to a decision to continue Iruplinalkib orally post-discharge.

At day 14, she visited our outpatient department. The hemoptysis had disappeared, and the previously severe chest pain was found to have completely resolved.

One month after Iruplinalkib initiation, a follow-up CT scan performed on September 27, 2024 revealed a dramatic reduction in the giant tumor (an 80.3% decrease from 132 mm to 26 mm), a notable reduction in the number of lung nodules, a significant shrinkage in the size of hilar and mediastinal lymph nodes, and a significant decrease in liver nodules (**Figure 1**). Additionally, she remained symptom-free and experienced no adverse drug reactions, with her body weight increasing by 3 kg (from 61 kg to 64 kg), indicating an excellent response to therapy.

Four months after Iruplinalkib initiation, a subsequent CT scan performed on December 11, 2024 demonstrated that the tumor had diminished compared to September 27, with the patient in good condition (**Figure 1**).

Six months after Iruplinalkib initiation, a subsequent CT scan performed on February 26, 2025 demonstrated that the giant tumor had nearly disappeared, with the patient continuing to exhibit no symptoms and no adverse reactions (**Figure 1**).

Nine months later, the patient’s follow-up CT on May 30, 2025 showed no significant changes compared to the previous scan, and the patient’s general condition was good (**Figure 1**).

Patient Perspective: At the most recent follow-up, the patient reported a significant improvement in her quality of life. She stated that the severe chest pain, hemoptysis, and cough that had initially troubled her completely resolved after initiating Iruplinalkib treatment and did not recur over the following nine months. No adverse events were experienced during the course of therapy. She also noted feeling more energetic and able to carry out daily activities without discomfort. The patient expressed satisfaction with the treatment outcome and gratitude for the support provided by the medical team. She emphasized that the relief of symptoms had a profoundly positive impact on both her physical and emotional well-being, as well as that of her family.

## Discussion

Iruplinalkib, a second-generation *ALK/ROS1* TKI developed by Qilu Pharmaceutical Co., Ltd., was approved in China in June 2023 (8). It inhibits *ALK* fusion proteins, blocking downstream signaling pathways such as ERK, STAT5 and AKT, thereby inducing tumor cell apoptosis. Optimized with a diphenylpyrimidine scaffold and four key modifications—a chlorine atom at the C5 position, a methoxy group at the C2′ position of the aniline, a methyl spirodiamine group at the C4′ position, and a dimethyl phosphonyl group at the C4 position of the aniline—Iruplinalkib shows enhanced potency against *ALK* fusions and resistance mutations (8). It offers better safety and efficacy, with an ORR and PFS surpassing other *ALK*-TKIs (10). Clinical studies demonstrate that iruplinalkib significantly extends PFS to 27.7 months compared to 14.6 months with crizotinib, with a hazard ratio (HR) of 0.34 (P<0.0001), indicating a 66% reduction in the risk of disease progression or death. The median duration of response was 26.78 months for iruplinalkib versus 12.88 months for crizotinib (10). However, current Phase I-III trials have only included Chinese patients, and iruplinalkib is not yet approved outside China. Research on its efficacy in other populations remains limited and unexplored (10, 11).

According to the 2023 edition of the Chinese Society of Clinical Oncology Guidelines for the Diagnosis and Treatment of NSCLC, Iruplinalkib is recommended as the preferred treatment following resistance to first-line *ALK*-TKI therapies in *ALK*–positive patients (8). In a single-arm, multicenter phase II clinical trial, the efficacy and safety of Iruplinalkib were evaluated in patients with advanced *ALK*-positive NSCLC who had developed resistance to crizotinib. A total of 146 patients received Iruplinalkib treatment. Results showed an ORR of 67.8% (95% CI: 59.6%–75.3%) and a disease control rate (DCR) of 96.6% (95% CI: 92.2%–98.9%). The median duration of response (DOR) was 13.1 months, and the median PFS was 14.4 months. Among these patients, 90 (61.6%) had brain metastases, and 41 (46%) had measurable intracranial lesions. The intracranial ORR with Iruplinalkib was 63% (95% CI: 47%–78%), indicating good intracranial disease control in patients with crizotinib-resistant brain metastases (12). As a novel *ALK/ROS1* TKI, Iruplinalkib is designed to overcome acquired resistance mutations to crizotinib, including the *ALK G1202R* mutation. The IC50 of Iruplinalkib against *ALK G1202R* mutant cells was 96 nM, demonstrating greater potency compared to alectinib (1000 nM) and brigatinib (340 nM). In addition, *in vitro* studies have shown that Iruplinalkib has strong inhibitory activity against various *ALK*-mutant cell lines, including those resistant to crizotinib and other *ALK* inhibitors, such as C1156Y, L1196M, F1174L/C/V, G1269A, I1171S/T/N, S1206F, V1180L, T1151Tins, and L1152R/P, with IC50 values ranging from 1.5 to 36.0 nM (8).

Diagnosed with advanced NSCLC and multiple metastases, the patient was treated with iruplinalkib following the detection of an *ALK* fusion mutation. Within one month, her tumor size dramatically reduced from 132 mm × 97 mm to 26 mm × 21 mm, with clinical symptoms significantly alleviated. By six months, the tumor had nearly vanished, showcasing iruplinalkib’s remarkable antitumor efficacy and potential to render cancer “reversible”. This study also has limitations. As a case report, one clear limitation is the fact that the evidence shown in this study should be confirmed in other patients. Iruplinalkib is approved only in China, therefore, there is no evidence of its use in other countries. Furthermore, it is important to consider whether the drug’s efficacy is associated with the patient’s age, ethnicity, and disease stage. To address these issues, we have designed a larger-sample clinical cohort study, which has already been approved by the Ethics Committee of Linyi People’s Hospital (clearance number: 202504-H-036) and formally initiated. Future work will explore the efficacy and safety of Iruplinalkib in other patients, as well as the correlation between age, genetic factors, and lung cancer outcomes. Additionally, given the patient’s age at the onset of lung cancer and her family history, we considered the possibility of hereditary cancer syndromes and recommended genetic counseling and next-generation sequencing (NGS) testing. Regrettably, the patient declined these tests due to financial constraints. In future studies and follow-ups, we plan to apply for relevant assistance programs to help her complete these evaluations.

Through this study, we demonstrated that Iruplinalkib has a remarkable tumor-shrinking effect, particularly in adolescent patients with *ALK*-positive lung cancer. The use of Iruplinalkib in this population may offer greater clinical benefit and could be considered as a first-line treatment option. We also advocate for the global adoption of Iruplinalkib to collect more clinical data and benefit a broader population of patients.

## Data Availability

The original contributions presented in the study are included in the article/**Supplementary Material**. Further inquiries can be directed to the corresponding authors.
