# Supplementary material for: Remarkable tumor response to Iruplinalkib in a 14-year-old girl with ALK-positive advanced metastatic non–small-cell lung cancer: a case report
Source: Front Oncol. 2025 Jul 25;15:1645580. doi: 10.3389/fonc.2025.1645580 (PMC12331475; doi:10.3389/fonc.2025.1645580)
Supplement: Supplementary file 1 [file DataSheet1.pdf]

Supplementary Materials-1

1. Immunohistochemical Detection of Tumor Cell Proliferation

(1) Slide Baking:Paraffin-embedded tissue sections were placed in an oven and baked for 2 hours.

(2) Dewaxing:The slides were sequentially immersed in the following solutions:

Xylene I for 15 minutes

Xylene II for 15 minutes

100% ethanol for 5 minutes

95% ethanol for 5 minutes

80% ethanol for 2 minutes

70% ethanol for 2 minutes

Then washed three times with PBS, 10 minutes each time.

(3) Antigen Retrieval:

Freshly prepared citrate buffer was brought to a boil, and the slides were placed in the buffer, then allowed to cool to room temperature. After antigen retrieval, the slides were washed with PBS three times for 10 minutes each.

(4) Blocking Endogenous Peroxidase:

3% hydrogen peroxide was added to completely cover the tissue, followed by incubation in a 37°C incubator for 30 minutes to block endogenous peroxidase activity and reduce non-specific staining. Slides should not be allowed to dry during this step. After blocking, slides were washed with PBS three times, 10 minutes each.

(5) Blocking Non-specific Binding:

Slides were incubated with 10% goat serum at room temperature for 30 minutes. After blocking, the serum was removed.

(6) Primary Antibody Incubation:

Primary antibodies diluted in PBS were added to fully cover the tissue sections. The slides were incubated at room temperature for 30 minutes, then transferred to 4°C overnight.

(7) Rewarming and Washing:

The next day, slides were brought to room temperature for 30 minutes, followed by three PBS washes, 10 minutes each.

(8) Secondary Antibody Incubation:

HRP-conjugated secondary antibody was added to cover the tissue completely, and incubated at room temperature for 1 hour. After incubation, slides were washed three times with PBS, 10 minutes each.

(9) DAB Staining and Counterstaining:

DAB solution was prepared at a 1:19 ratio and applied to the slides. The staining process was monitored under a microscope, and stopped in time using tap water. After DAB staining, hematoxylin was applied for counterstaining. The process was again observed under a microscope and stopped as appropriate. Sections were then treated with ammonia water to blue the nuclei and washed immediately with tap water.

(10) Dehydration and Mounting:

Slides were dehydrated sequentially:

70% ethanol for 5 minutes
80% ethanol for 5 minutes
95% ethanol I for 5 minutes
95% ethanol II for 5 minutes
100% ethanol I for 15 minutes
100% ethanol II for 15 minutes
Xylene I for 15 minutes
Xylene II for 15 minutes
Finally, slides were mounted with neutral resin for long-term preservation.

Includes a Chinese version：
1.免疫组化检测肿瘤细胞的增殖
（1）烤片：将石蜡切片放入烘箱中烤片 2h。
（2）脱蜡：取出石蜡切片后依次浸入二甲苯Ⅰ 15min→二甲苯Ⅱ 15min→ 100%乙醇 5min → 95%乙醇 5min → 80%乙醇 2min → 70%乙醇 2min，PBS 洗三次，每次 10min。
（3）抗原修复：将新鲜配置的枸橼酸盐缓冲液煮沸，把玻片放进缓冲液中，放凉至室温，修复完成后，PBS 洗三遍，每次 10min。
（4）阻断内源性过氧化物酶：滴加 3%过氧化氢充分覆盖组织，37℃培养箱孵育 30min 以阻断内源性过氧化物酶，来降低非特异性沾染。操作过程中，应避免干片。阻断结束后，PBS 洗三次，每次 10min。
（5）封闭：加入 10%山羊血清室温封闭 30min，封闭结束后，倾去抗体。
（6）孵育抗体：加入用 PBS 稀释好的抗体，抗体应充分覆盖组织，室温放置 30min 后，4℃冰箱孵育过夜。
（7）第二天，取出玻片，放置于室内复温 30min，复温结束后，PBS 洗三次，每次 10min。
（8）滴加 HRP 标记的二抗，使之充分覆盖组织，室温孵育 1h。孵育结束后，PBS 洗三次，每次 10min。
（9）DAB 显色：按照 1:19 的比例配置好 DAB 显色液后，镜下观察显色结果，及时用自来水终止显色反应。显色结束后，滴加苏木素，复染，镜下观察染色结果，及时终止染色。染色结束后，用氨水反蓝，结束后立即用自来水冲洗。
（10）脱水：70%乙醇 5min→ 80%乙醇 5min → 95%乙醇Ⅰ 5min → 95%乙醇Ⅱ 5min →100%乙醇Ⅰ 15min → 100%乙醇Ⅱ 15min→二甲苯Ⅰ 15min→二甲苯Ⅱ 15min。脱水结束后，中性树胶封片保存。

2.
